# HIV-positive and HHV-8-negative primary effusion lymphoma complicated with coronary heart disease: a Case Report and literature review

**DOI:** 10.3389/fmed.2025.1527960

**Published:** 2025-08-26

**Authors:** Wei Zhang, Ying Zhang, Jing Yuan

**Affiliations:** ^1^Division of Infectious Diseases, Chongqing Public Health Medical Center, Chongqing, China; ^2^Department of Pathology, Chongqing, China

**Keywords:** acquired immune deficiency syndrome (AIDS), HHV8-negative, primary effusion lymphoma (pel), coronary heart disease (CHD), HIV

## Abstract

**Objective:**

This case report describes a rare instance of human immunodeficiency virus (HIV)-positive, human herpesvirus-8 (HHV-8)-negative, and Epstein-Barr virus (EBV)-negative primary effusion lymphoma (PEL) associated with pulmonary effusions likely caused by congestive heart failure (CHF). The new classification added in the fifth edition of the World Health Organization’s lymphoma classification is named “Fluid Overload-Associated Large B-Cell Lymphoma.”

**Methods:**

The diagnosis and treatment of a 71-years-old male patient with acquired immune deficiency syndrome (AIDS) and PEL admitted to the Department of Infectious Diseases in Chongqing Public Health Medical Center in March 2022 were detailed, and relevant literature was reviewed.

**Results:**

The patient had been receiving antiretroviral therapy (ART) with lamivudine (3TC) + tenofovir (TDF) + efavirenz (EFV) for 17 years. The primary clinical manifestations were large pericardial and pleural effusions. Pathological examination confirmed HHV-8 and EBV-negative large B-cell lymphoma. The effusions were drained effectively, and the patient received ART and two cycles of R-CDOP chemotherapy. Atrial fibrillation and coronary heart disease (CHD) were diagnosed during the course of the disease. After 2 years of follow-up, no recurrence of symptoms was observed.

**Conclusion:**

This case highlights the importance of recognizing rare HHV-8 negative PELs and the need to monitor underlying conditions such as CHD that may contribute to effusion formation. It underscores the diagnostic challenges and the necessity of a multidisciplinary approach in managing such cases.

## 1 Introduction

Primary effusion lymphoma (PEL) is a rare non-Hodgkin lymphoma that is referred to as a large B-cell lymphoma without any detectable tumor mass in the fifth edition of the WHO (1). PEL mainly manifests as effusion in body cavities such as the thoracic, abdominal, and pericardial cavities. Human herpes virus-8 (HHV-8) was positive in all classic cases of PEL (2, 3). Most cases are acquired immune deficiency syndrome (AIDS)-related cancers, and the clinical manifestations are due to immunodeficiency (4). In the past decade, HHV8-negative human immunodeficiency virus (HIV)-negative PEL cases have been reported. We present a case report of a patient with HIV-positive, HHV-8-negative, and Epstein-Barr virus (EBV)-negative PEL who was diagnosed and treated at the Chongqing Public Health Medical Center. The patient was diagnosed with coronary heart disease (CHD) during hospitalization. The patient was managed with drainage of the effusion, effective anti-HIV therapy, two sets of chemotherapy, and secondary preventive measures for CHD. After 2 years of follow-up, no recurrence of PEL was observed. Reviewing the patient’s medical history and immunohistochemical markers, in accordance with the newly added classification of lymphomas in the fifth edition of the WHO, it was named as fluid overload-related large B-cell lymphoma (1).

## 2 Case presentation

A 71-years-old married male with HIV infection since 2005 was on antiretroviral therapy (ART) with lamivudine (3TC), tenofovir (TDF), and efavirenz (EFV) without baseline CD4 and HIV-RNA measurements.

**FIGURE 1:**
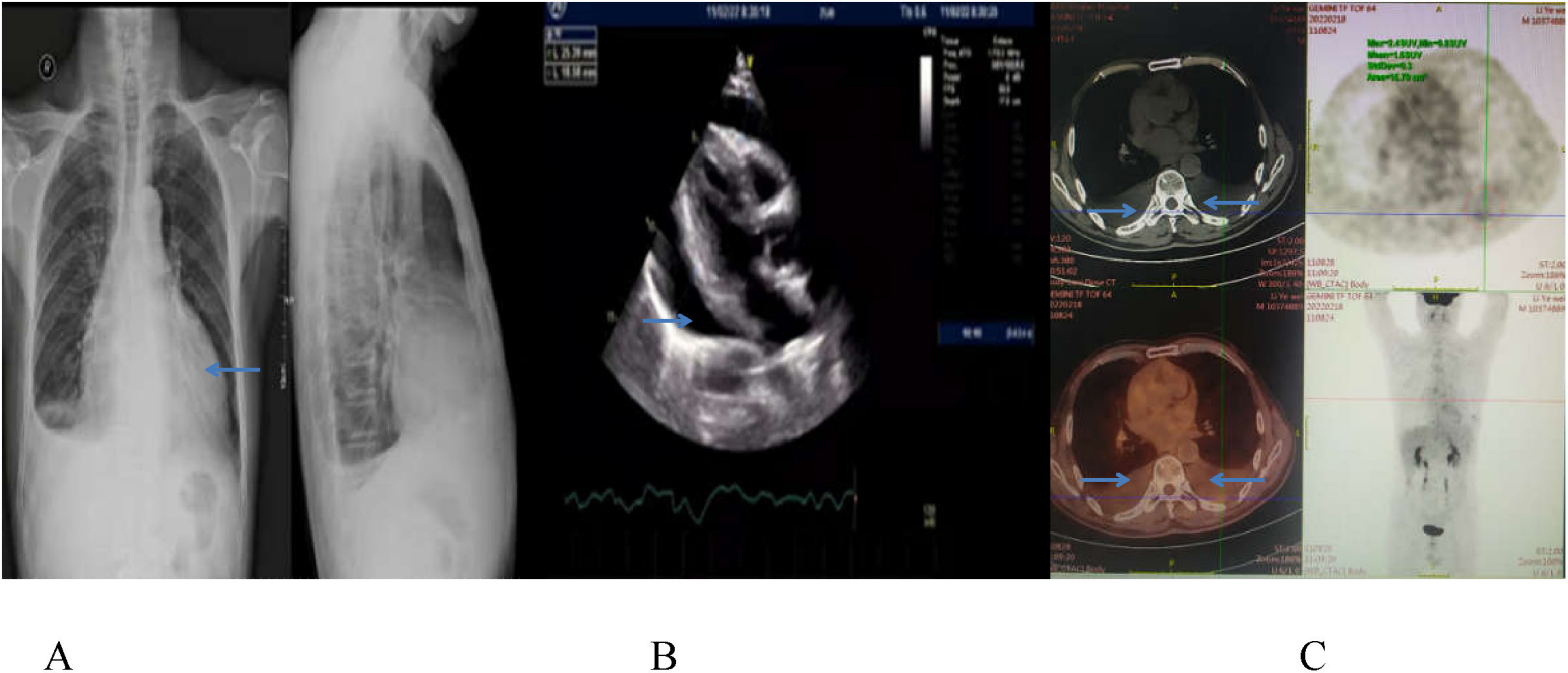
Chest CT image **(A)** and echocardiographic image **(B)** show a large amount of pericardial fluid on February 11, 2022, and PET-CT scan **(C)** displays pericardial and bilateral pleural effusion, with slightly increased FDG metabolism, and no space-occupying lesion on February 18, 2022.

On February 11, 2022, he was admitted to a local hospital due to dyspnea. Imaging revealed significant pericardial and pleural effusions, no obvious lumps or enlarged lymph nodes were found (Figures 1A–C). Pericardiocentesis drained approximately 600 mL of bloody pericardial effusion, and lymphoma was suspected on pathological examination.

**FIGURE 2:**
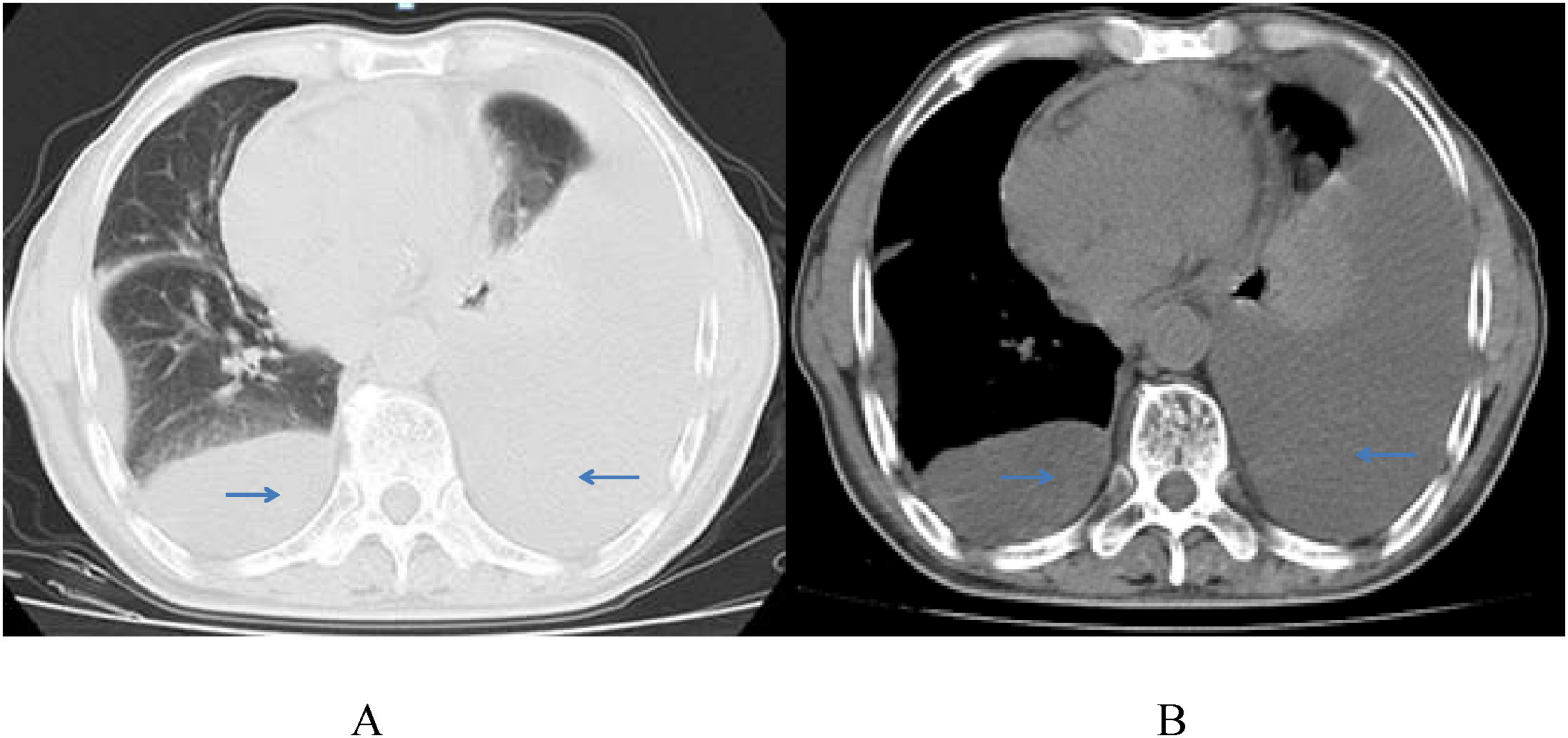
Chest CT scans **(A,B)** show a large amount of bilateral pleural effusion, partial atelectasis of the left lung, pericarditis, and no pleural space-occupying lesion on March 22, 2022.

On March 18, 2022, the patient was readmitted to our center with recurrent dyspnea, chest tightness, and an 8 kg weight loss. Physical examination revealed low heart sounds, enlarged cardiac silhouette, a heart rate of 118 beats per minute, and an irregular heartbeat; no valve murmurs were heard on auscultation, and no enlarged lymph nodes were palpable. Laboratory tests showed a low CD4 count of 191 cells/μL, an undetectable HIV viral load (<50 copies/ml), elevated lactate dehydrogenase (LDH, 651 U/L), troponin T of 0.0200 μg/L, myoglobin content of 8.54 ng/mL, B-type natriuretic peptide of 177 pg/mL, and normal renal and hepatic function. Chest CT revealed bilateral massive pleural effusion and pericarditis (Figure 2). Closed thoracic drainage was performed, yielding approximately 4500 mL of pleural effusion.

Pleural fluid analysis revealed a bloody and turbid appearance with a positive Rivalta test. The white blood cell (WBC) count was 21,126 × 10^6^/L, with 20,839 × 10^6^/L mononuclear cells and 287 × 10^6^/L polymorphonuclear cells. Red blood cell (RBC) count was 143 × 10^9^/L. Biochemical analysis showed lactate dehydrogenase (LDH) at 6281 U/L, adenylate deaminase (ADA) at 62.5 U/L, and total protein at 52.5 g/L. No pathogenic bacteria, fungi, or *Mycobacterium tuberculosis* were detected.

**FIGURE 3:**
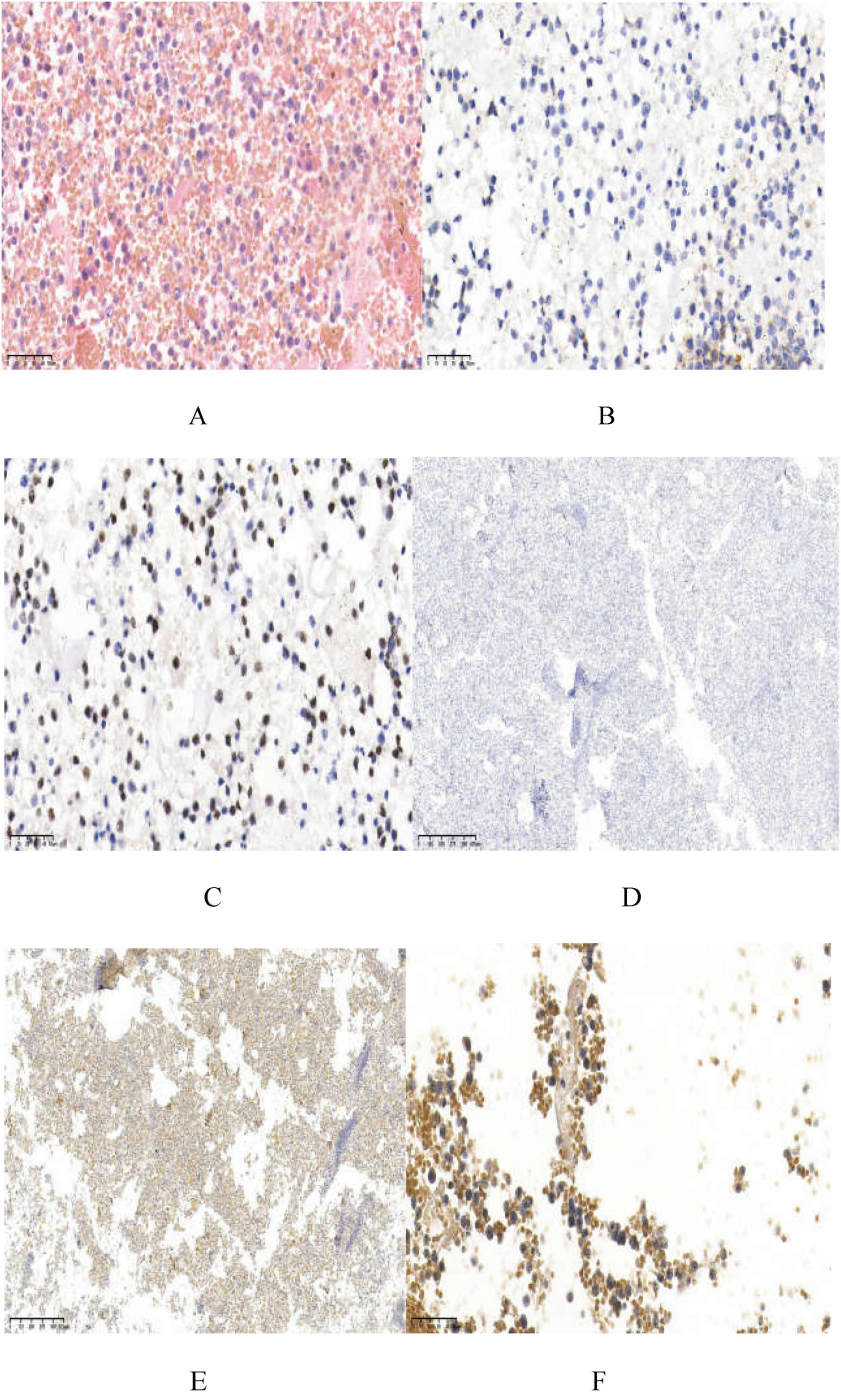
Staining of the tumor specimen. **(A)** Displays a large number of atypical lymphoid cells diffuse infiltration, medium-sized tumor cells, diffusely growing enlarged nuclei (HE; ×400); **(B,C)** show CD20 positive (×400) and PAX-5 positive (×400); **(D)** shows HHV8-negative (×40); **(E)** displays EBER-negative (×40); **(F)** has C-myc positive cells, about 75% (×400).

Immunohistochemical staining of the pleural and pericardial effusions showed tumor cells positive for CD20, CD10 (approximately 10%), Mum-1 (approximately 80%), Bcl-2 (approximately 10%), CD79α, PAX-5, CD138, and C-myc (approximately 75%), and negative for CD21, CD38, HHV8, CD30, CD5, CD3, CK-pan, TTF1, Syn, CgA, and CD56. The Ki-67 proliferation index was estimated at 50%–60%. *In situ* hybridization for EBER and HHV8 was negative. Pathological evaluation confirmed HIV-associated diffuse large B-cell lymphoma, non-GCB type by Hans classification (Figure 3).

Based on the findings, the patient was diagnosed with diffuse large B-cell lymphoma (DLBCL) associated with acquired immune deficiency syndrome (AIDS). On March 30, 2022, the patient received R-CDOP chemotherapy (cyclophosphamide 750 mg/m^2^ on day 1; liposomal doxorubicin 30 mg/m^2^ on day 1; vincristine 1.4 mg/m^2^ on day 1, with a maximum dose of 2 mg; prednisone 100 mg on days 1–5). Due to drug interactions and adverse reactions, the antiretroviral therapy (ART) regimen was changed to 3TC/TDF/ABT, resulting in significant improvement and subsequent discharge.

**FIGURE 4:**
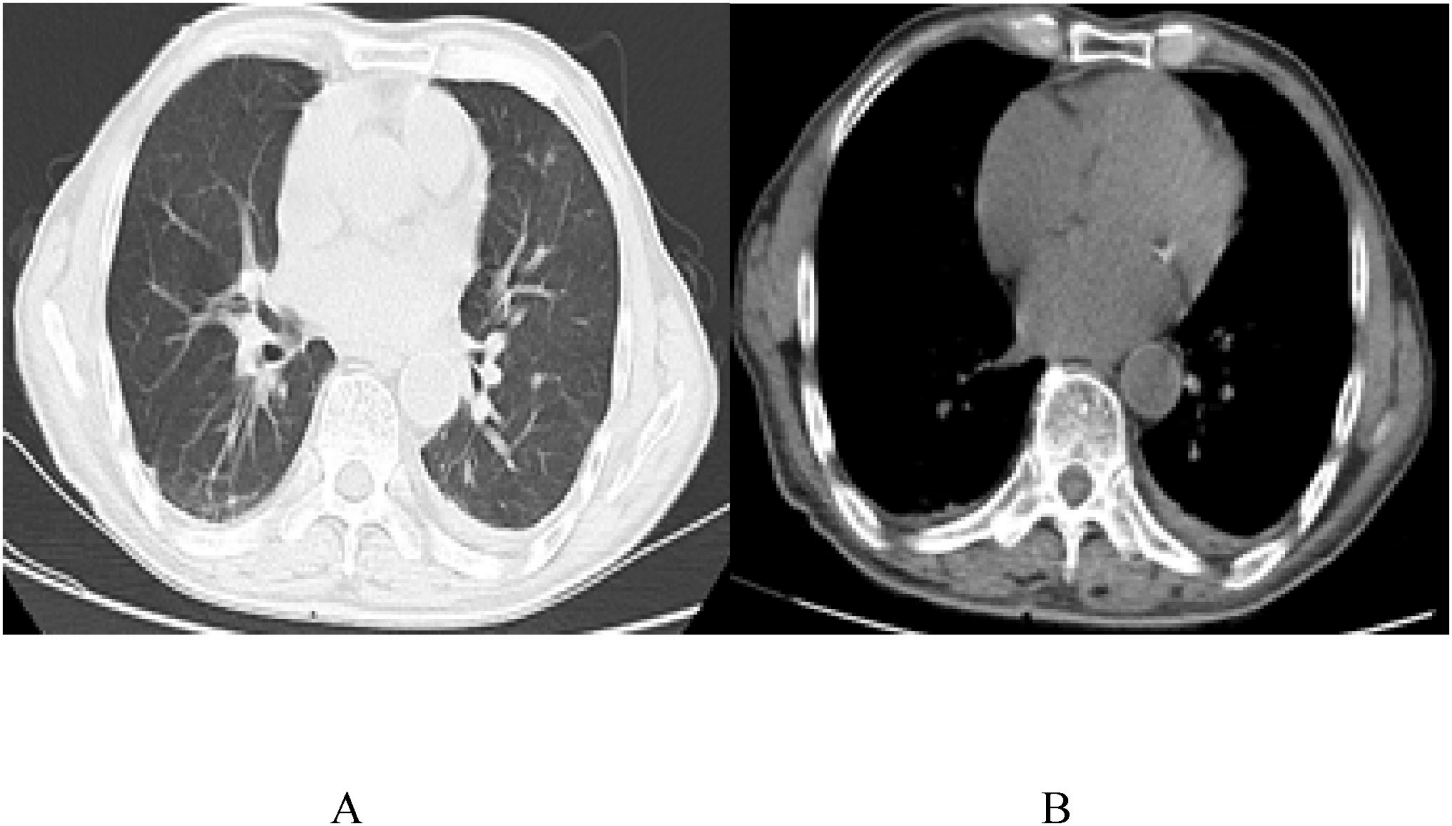
Chest CT images **(A,B)** on the reexamination 6 months after the diagnosis, on September 29, 2022, with no pleural and pericardial effusions.

On April 25, 2022, the patient was readmitted with cough. Reexamination showed a CD4 count of 174 cells/μL and an HIV-RNA load of < 20 copies/mL. Echocardiography revealed a small amount of pericardial effusion, while chest CT showed a significant increase in bilateral lung lesions. *Pseudomonas aeruginosa* was detected in the bronchoalveolar lavage fluid using mNGS, with 814 reads. After anti-bacterial treatment (Piperacillin Tazobactam Sodium), the second cycle of R-CDOP chemotherapy was administered. The patient experienced sudden acute heart failure and rapid atrial fibrillation. Coronary CTA revealed mild to severe stenosis in the middle and distal segments of the left anterior descending branch (20%–85% stenosis), consistent with coronary atherosclerosis. The patient was treated with secondary prevention drugs for coronary heart disease (CHD) but refused further chemotherapy due to complications and was discharged. The patient had CHD complications and was treated with secondary prevention drugs. On September 29, 2022, a chest CT revealed no pleural or pericardial effusion (Figure 4). The patient is still being followed-up without any recorded complaints (Table 1).

**TABLE 1:** Timeline.

Timeline	
2005	Diagnosed with AIDS, started 3TC/TDF/EFV anti-HIV treatment
11 February, 2022	The patient was admitted due to dyspnea. Imaging showed pericardial and pleural effusions, and pericardiocentesis revealed bloody fluid with suspected lymphoma
18 March, 2022	*In situ* hybridization for EBER and HHV8 was negative, pathological evaluation confirmed HIV-associated diffuse large B-cell lymphoma, non-GCB type by Hans classification
30 March, 2022	The patient received R-CDOP chemotherapy and had his antiretroviral therapy (ART) regimen changed to 3TC/TDF/ABT
25 April, 2022	The patient was readmitted with cough and pericardial effusion, and *Pseudomonas aeruginosa* was detected. He received antibacterial treatment and R-CDOP chemotherapy He then experienced acute heart failure and coronary artery stenosis. He was treated for coronary heart disease but refused further chemotherapy and was discharged
29 September, 2022	CT revealed no pleural or pericardial effusion
Two years after discharge	No recurrence was observed

## 3 Discussion

Primary effusion lymphoma (PEL) is a rare invasive B-cell malignant tumor that accounts for 1%–4% of AIDS-associated lymphomas (5) and is commonly associated with HHV8 and EBV infections (6). PEL without EBV infection occurs mostly in elderly HIV-negative patients. The median age of onset in HIV-infected patients was 42 years and that in HIV-free patients was 73 years (7), with no significant sex difference. The case reports published in the PubMed database for the last 10 years (2012–2022) were retrieved using the keywords of “Human Herpesvirus 8-unrelated and Primary Effusion Lymphoma-Like Lymphoma.” Duplicated literature was excluded, the selected literature was carefully reviewed, and relevant data were collected for the analysis and synthesis of a summary. A total of 12 articles were retrieved, including 15 cases of HHV-8-negative PEL in which there were no HIV-positive patients (Table 1). To the best of our knowledge, this is a rare case of HIV-positive and HHV8-unrelated PEL. This type is characterized by large B-cell lymphoma with excessive body fluid according to the fifth edition of the WHO Health Organization lymphoma classification (1).

This case involved a 71-years-old man who had been diagnosed with HIV for 17 years and was receiving regular anti-HIV therapy. The main manifestation was fluid overload with massive bloody effusion in the pericardial and bilateral thoracic cavities. The patient had no high-risk conditions, such as hypertension, diabetes, hyperlipidemia, or smoking habits. Rapid atrial fibrillation occurred during treatment. Echocardiography revealed no obvious abnormalities in heart structure. Complete coronary CTA revealed symptoms of coronary atherosclerosis, and CHD was considered. These symptoms were consistent with previous reports on HHV8-unrelated PEL that usually occurred in elderly patients with underlying conditions of excessive body fluid effusion, such as CHD (8). Among the other reported cases selected for this review, 73.33% (11/15) of the patients had an onset age of ≥70 years, and 40% (6/15) had cardiovascular disease, liver cirrhosis, and other underlying conditions that may have caused body fluid overload. The most commonly affected sites were the pleural cavity (73.33%; 11/15) and pericardial cavity (33.33%; 5/15), which was similar to the present case. However, as shown in Table 2, HHV8-negative PEL mostly occurred in HIV-negative elderly patients with normal immune function (9), which differed from this HIV-positive patient.

**TABLE 2:** Characteristics of 15 cases with human herpesvirus 8-unrelated PEL-like lymphomas.

Case no	Reference	Age (y)/sex	Other disease conditions	HIV	EBV	HCV	HBV	Site of PEL	IHC	Therapy	Outcome
1	Aota et al. (16)	79/male	CAD, MDS	−	−	−	−	Peritoneum	CD10−, CD19+, CD20+	Glucocorticoid, drainage	Died, 0.75 months
2	Klepfish A et al. (17)	78/male	Hypertension, CVA	−	ND	ND	ND	Pleura	CD45RB+, CD20+, CD3−	CHOP	Died, 18 months
3	Chen et al. (18)	61/male	Alcoholic cirrhosis	−	+	−	−	Pleura, pericardium	CD138+, MUM-1+, EMA+, CD3−, CD30−, CD45−, CD19−, CD20−, CD79a−, PAX5−	None	Died, 0.5 months
4	Xiao et al. (19)	55/male	Alcoholic cirrhosis	−	−	−	−	Pleura, pericardium	ND	None	Died, 4 months
5	Xiao et al. (19)	95/male	Hypertension, basal cell carcinoma, atrial fibrillation	−	−	−	−	Pleura	CD19+, CD20+, CD22+, CD10+, CD5−, CD23−, CD38−	Rituximab, drainage	Alive, 72 months
6	Xiao et al. (19)	69/Female	Thyroid cancer	−	−	ND	ND	Pericardium	CD19 + , CD20 + , CD38 +	R-CHOP	Alive, ND
7	Ogasawara et al. (20)	89/female	ND	−	−	ND	ND	Pleura	CD19+, CD20+, CD10+, CD38+, CD7+, BCL2+, BCL6+, CD5−, CD30−, MUM1−	Etoposide, R	Died, 13 months
8	Kim et al. (21)	73/male	ND	−	−	ND	ND	Pericardium	ND	R-CHOP	Died, 3 months
9	Kojima et al. (22)	69/male	CML	−	−	−	−	Pleura	CD20+, MUM-1+, CD10+, BCL2+, BCL6+, CD79a+, CD3−, CD5−, CD7−	R-CHOP, R-CVP	ND
10	Kidoguchi et al. (23)	97/female	Osteoporosis	−	−	−	−	Pleura	CD3+, CD4+, CD30+, CXCL13+, CD8−, CD10−, CD20, Bcl-6, PAX5, PD-1	Glucocorticoid	ND
11	Horiuchi et al. (24)	74/female	ND	−	ND	ND	ND	Pericardium	ND	Drainage	Alive, 48 months
12	Nakatsuka et al. (25)	70/male	ND	−	ND	ND	ND	Pleura	CD3−, CD20+, CD79a+, CD138−	Drainage	Alive, 14 months
13	Nakatsuka et al. (25)	70/male	ND	−	ND	ND	ND	Pleura	CD20+, CD45RO−, CD138−	Drainage	Alive, 25 months
14	Shin et al. (26)	77/male	ND	−	−	−	−	Pleura	CD45+, CD20+, MUM-1+, CD138−, CD79a−, CD3−, Bcl-2−, Bcl-6−, Ki67: 90%	R-CHOP	Alive, 15 months
15	Toyoda et al. (27)	70/male	Cardiac	−	ND	ND	ND	Pleura	CD20+	R, Etoposide	Alive, 7 months

CAD, coronary artery disease; MDS, myelodysplastic syndrome; CVA, Cough variant asthma; ND, not done or not described; R-CHOP, rituximab, cyclophosphamide, doxorubicin, vincristine, and prednisolone; R-CVP, rituximab, cyclophosphamide, vincristine, and prednisolone.

Pathological examination of the effusion fluid revealed a large number of atypical lymphoid cells with diffuse infiltration, medium-to large-sized cells, round or oval slightly irregular nuclei, rough chromatin, visible nucleoli, and obvious mitosis. B cell markers (CD20, CD79a, and PAX-5) were positive in the tumor cells. According to the Hans model (10), this case was a non-germinal center B-cell-like subtype (10%–30% CD10 positive; MUM-1 positive) and lacked the expression of the plasmablastic phenotype (CD45 and CD30) from the classical PEL. These neoplastic cells frequently exhibit plasmablastic differentiation, typically expressing plasma cell-associated antigens such as CD38, CD138, and VS38c. Of particular therapeutic significance, the consistent absence of CD20 expression in PEL. Typical PEL caused by B cells infected with HHV8 (65%–80%) is often co-infected with EBV (1), indicating the synergistic effect of EBV and HHV8 in the pathogenesis of PEL. However, the effusion in this case was HHV-8-negative and EBER-negative by *in situ* hybridization labeling, which was inconsistent with the HHV-8-positive and EBV-positive PEL reported in HIV-positive individuals. The patient had a rare form, and the mechanism was unknown (11).

There is no standard treatment for large B-cell lymphomas with excessive body fluid effusion. CHOP-containing cyclophosphamide, doxorubicin, vincristine, and prednisone chemotherapy has been used in most reported cases. As tumor cells often express CD20, treatment with rituximab is superior to classic PEL treatment. In these previous studies, some patients had drainage alone (15/34; 44%), some patients received rituximab only (4/34; 12%) or chemotherapy alone (15/34; 44%), and the median overall survival (OS) was 14.9 months (12). The patient received two cycles of R-CDOP chemotherapy and refused further targeted antitumor therapy. However, the effusion fluid was completely absorbed, and the patient had no recurrence of symptoms for up to 2 years of follow-up.

Antiretroviral therapy (ART) is essential for treating HIV-positive PEL. Owing to drug interactions, bone marrow suppression after chemotherapy, gastrointestinal reactions, liver and kidney injury, and other side effects, an effective antiviral therapy was selected using the fusion inhibitor albuvirtide (ABT). The patient’s viral load was controlled throughout the follow-up period. In a previous report, combined antiretroviral therapy significantly changed the life expectancy of patients with HIV infection and lymphoma (13). In HIV-positive PEL, the survival of patients receiving ART is prolonged (14). Therefore, regardless of whether the patient is on ART before the diagnosis of PEL, ART should be initiated as soon as possible after the diagnosis. The patient received secondary therapy for CHD, which may have been a risk factor for excessive bodily fluid production. This was consistent with a previous report (15) that treatment of underlying conditions causing excessive body fluid effusion in HHV8-negative PEL improved the prognosis of patients.

In the cases reported in the past 10 years, 53.33% (8/15) of patients with HHV8-unrelated PEL who were treated with drainage alone or combined chemotherapy survived for more than 6 months, with an average survival of 18.35 ± 21.44 months. The possible reasons for the overall good prognosis in this case might have been related to the treatment of the underlying conditions, including ART and secondary therapy for CHD.

## 4 Conclusion

In conclusion, this report presents a case of HHV-8 negative PEL with CHD in an AIDS patient, which is clinically rare. PEL was caused by the invasion of lymphoma into the body cavity, but there was no lump. In HIV-positive individuals, HHV8-positive and HHV8-negative PELs show similar clinical characteristics. However, monitoring PEL for other underlying conditions causing excessive body fluid effusion and different cellular morphologies and immunophenotypes is the key to correct diagnosis and management. In patients with HIV/AIDS, effective anti-HIV therapy should be combined with continuous fluid drainage. For the treatment of underlying conditions that cause excessive body fluid production, multidrug chemotherapy consisting of rituximab may prolong the survival of patients with large B-cell lymphoma with excessive body fluid effusion.

## Data Availability

The raw data supporting the conclusions of this article will be made available by the authors, without undue reservation.
